# Editorial: Model organisms in plant science: Maize

**DOI:** 10.3389/fpls.2023.1147857

**Published:** 2023-02-08

**Authors:** Ana Butrón, Rogelio Santiago, Manje Gowda

**Affiliations:** ^1^ Misión Biológica de Galicia, Spanish Council for Scientific Research (MBG-CSIC), Pontevedra, Spain; ^2^ Agrobiología Ambiental, Calidad de Suelos y Plantas University of Vigo (UVIGO), Unidad Asociada a la MBG (CSIC), Vigo, Spain; ^3^ Global Maize Program, International Maize and Wheat Improvement Center (CIMMYT), Nairobi, Kenya

**Keywords:** *Zea mays* L., maize, model organism, plant physiology, plant breeding, heterosis, genomic selection

Maize has been an organism of historical importance to all biologists as eminent researchers such as Beadle, Emerson, McClintock, Stadler and Rhoades made ground-breaking genetic discoveries in maize that hold true for all living organisms (Andorf et al., 2016). Nowadays, plant lignocellulose represents the world’s greatest repository of renewable energy amenable to conversion into liquid, and maize has become one of the preferred choices due to their high biomass yields, broad geographic adaptation, carbon sequestration potential and nutrient utilization (Courtial et al., 2013; van der Weijde et al.). Therefore, this Research Topic aimed (1) to put forward the importance of research focuses in maize as a model organism, presenting recent developments and important accomplishments in moving forward the study of plants, and (2) to shed light on the progress made in the past decade working with maize as an important crop used worldwide.

Today, plant genome editing using clustered regularly interspaced short palindromic repeats (CRISPR)/CRISPR-associated protein 9 (Cas9)) technology has rapidly become the preferred tool to generate mutants for functional genomics in plants. Despite their rapid success, plant genome editing is still a labour-intensive process. Fierlej et al. presented an optimized maize protoplast system and a specifically developed bioinformatics pipeline to evaluate rapidly the efficiency of CRISPR/Cas9 constructs or of novel Cas9 variants before engaging in time- and resource-consuming stable transformation. This protocol is useful in improving maize genome editing processes which ultimately improve its success rate. This knowledge can be meaningful for breeding programs.

Another paper that makes a direct contribution to plant breeding practice has been written by Ni et al. These authors show that the use of correlated traits and sparse phenotyping can yield high prediction accuracies while reducing the cost of extensively phenotyping for difficult to measure traits like kernel water content at black layer formation. Recommended methodological approaches although applied to a relevant trait for maize breeding could be useful for performing genomic selection for traits that are difficult to measure/record in any other plant organism.

In the same way, five other research papers and one corrigendum that deal with fundamental aspects of plant breeding were accepted. Paper by Sang et al. contributed to a better comprehension of heterosis; this phenomena along with transgressive segregation being the reasons that plant breeding works (Mackay et al., 2021). Sang et al. show that epistasis contribution to heterosis is not negligible because the interaction of many minor-effect genes in the hybrids could activate the transcription activators of epistatic genes, resulting in a cascade of amplified yield heterosis. Authors also provide recommendations to accelerate hybrid maize breeding by activating epistatic effects.

Zhang et al. in a research paper and a corrigendum provide important findings allowing further elucidation of the molecular mechanism of regulation of leaf angle. Leaf angle is closely associated with canopy structure and photosynthetic efficiency under high planting density and have become crucial for improving yield. The leaf angle regulation functions of bHLH TF have been studied in rice, but important questions remain unsolved. Authors compare the transcriptome of a *Zmbhlh112* mutant, which displays a relatively small leaf angle, and the wild-type B73, and found that ribosomal subunits may play an important role in leaf development.

Li et al. also focuses in the genetic control of traits related to grain yield as ear fascination and tillering. Although most of the modern maize hybrid cultivars cultivated in the high-dense stands in temperate environment develop only one ear, the potential presence of multiple ears per plant has physiological and breeding implications. The fasciation effects and the correlated effect on kernel row number were confirmed across genetic backgrounds, making the QTL identified an interesting source of yield-positive alleles. Unexpectedly, authors did not find correlation or QTL overlaps between ear prolificacy and tillering, although these traits share a developmental basis.

On the other hand, due to climate change, plants need to adapt more quickly under unpredictable climate change scenarios. Low temperature effect on germination of maize seedlings is critical to have good germination. For eight low-temperature resistance related traits Zhou et al. identified twenty QTL, of which seven QTL overlapped in single region on chromosome 1 at 197-202 Mb named as cQTL1-2. QTL identified on this region explained 5 to 26% of total phenotypic variation and were consistent with previous studies on low temperature resistance QTL. Two candidate genes identified in this region, GRMZM2G082630 and GRMZM2G115730, were upregulated in low-temperature tolerant lines. The authors proposed these candidate genes can be exploited through breeding to improve the low-temperature tolerance during seedlings germination.

Maize shows significant variation for salinity tolerance which encourages the researchers to identify and understand the genetic architecture of salinity tolerance. Root system plays an important role in salinity tolerance and lateral roots are important for water and nutrient acquisition. Zhang et al. planned experiment to identify the genetic basis of natural variation in lateral root length which is of great agronomic relevance to improve salt tolerance. Through GWAS, authors identified the causative gene *ZmSULTR3:4*, which encodes a plasma membrane-localized sulfate transporter and is associated with natural variation in maize lateral root length under salt stress. Overall, authors conclude the *ZmSULTR3:4* gene has regulatory role in lateral root growth which can be used to improve maize root traits and salinity tolerance by molecular breeding.

As previously mentioned, drought and heat stresses are major limiting factors for crop growth and productivity (Fahad et al.), and they cause the greatest annual loss of crops (Ray et al., 2015). Plants have developed multiple responses at the developmental, physiological, and molecular levels that enable them to escape, avoid, and/or tolerate unfavorable environmental conditions (Chávez-Arias et al.). From a physiological point of view, an interesting mini review paper by Serna explores how maize yield may persevere through climate change by focusing on the stomatal regulation of gas exchange. The paper reviews the maize stomatal response to drought and heat stresses and the possible molecular mechanisms of maize stomatal development in response to climate change. Under drought stress, maize respond by increasing abscisic acid levels and thereby reducing stomatal opening (Zhao et al., 2015), whereas author put forward that in order to avoid plant heat damage, maize could increase the number of stomatal files and, consequently, the stomatal density, by expanding the expression domain of *ZmSHR1*.

The accepted articles demonstrate how cutting-edge approaches in maize genetics and breeding are useful in crop improvement. The contributions of authors are of highest quality and illuminate the strong international interest in this Research Topic. These results would help maize researchers, geneticists, and breeders in order to improve the understanding on maize genetics and contribute to increase maize yield under diverse agroclimatic conditions.

## Author contributions

AB, RS, and MG contributed to the writing of this editorial. All authors revised and improved the final version of the editorial.
